# The role of subjective interoception in autobiographical deficits in aphantasia

**DOI:** 10.1038/s41598-025-23270-x

**Published:** 2025-12-01

**Authors:** Merlin Monzel, Yoko Nagai, Juha Silvanto

**Affiliations:** 1 Department of Psychology, Personality Psychology and Biological Psychology, University of Bonn, Bonn, Germany; 2 College of Medicine and Veterinary Medicine, The University of Edinburgh, Edinburgh, Scotland; 3 Department of Clinical Neuroscience, Brighton and Sussex Medical School, University of Sussex, Sussex, UK; 4 School of Psychology, Faculty of Health and Medical Sciences, University of Surrey, Surrey, UK; 5 Centre for Cognitive and Brain Sciences, University of Macau, Taipa, China; 6 Department of Psychology, Faculty of Health Sciences, University of Macau, Taipa, China

**Keywords:** Mental imagery, Interoception, Autobiographical memory, Episodic details, Aphantasia, Hypophantasia, Psychology, Human behaviour

## Abstract

Autobiographical memory deficits are well-documented in aphantasia, yet the underlying mechanisms remain unclear. Emerging models suggest that interoception plays a crucial role in mental imagery, a key component of memory retrieval. In this study, we investigate the relationship between self-reported interoception, mental imagery, and autobiographical memory, with a specific focus on aphantasia. First, we examined whether interoceptive awareness and autobiographical memory differ between individuals with core aphantasia (*n* = 69), hypophantasia (*n* = 266) and typical imagers (*n* = 133). Our findings reveal that aphantasics report significantly lower autobiographical memory as well as subjective interoceptive awareness across key subscales, including emotional awareness and noticing. Secondly, a mediation analysis reveals that mental imagery mediates the relationship between the emotional awareness subscale of the Multidimensional Assessment of Interoceptive Awareness (MAIA) questionnaire and autobiographical memory, suggesting that subjective interoception may contribute to memory recall indirectly through its influence on imagery. These findings provide novel empirical support for the idea that interoception is linked to both mental imagery and memory retrieval. The reduced interoceptive awareness observed in aphantasia may contribute to their known deficits in autobiographical memory, positioning aphantasia as a condition that extends beyond a lack of mental imagery to include altered interoceptive processing.

## Introduction

Aphantasia denotes ‘the absence, or near-absence, of imagery’^1^. Since the term was coined in 2015, many studies have investigated the consequences of aphantasia on several cognitive processes, such as mental rotation^2,3^, attentional guidance^4,5^, or face recognition^6–8^. One of the most robust associations was found between aphantasia and a reduced richness of episodic details in autobiographical memory^9–11^, which has also been linked to SDAM (severely deficient autobiographical memory)^12,13^. Since autobiographical imagery deficits are associated with considerable distress in aphantasia^14^, it is important to understand the underlying mechanisms, ultimately enabling us to develop compensatory strategies for aphantasics. Aim of the present study is thus to explore the role of self-reported interoceptive abilities in the formation of autobiographical memories due to recent findings that interoceptive abilities are reduced in aphantasics^15^.

In a 2023 study, one aphantasic participant reported: “I have almost no memory of past events, even important ones like my wedding and the births of our children”^14^. Building on this and similar case reports^16^, evidence for autobiographical memory impairments in aphantasics was found in several studies, using a variety of measures. For example, Zeman et al. asked their participants to indicate how good their autobiographical memory was, leading to over 30% of aphantasics responding with “bad” in contrast to less than 10% of controls^17^. Following this, Dawes et al., Milton et al. and Monzel et al. applied the Autobiographical Interview by Levine et al.^18^ to assess the richness of episodic details in aphantasics’ autobiographical memory recall^9–11^. Consistently, they found less episodic details for memory reports of aphantasics compared to controls, whereas non-episodic memory recall was not affected. Similar results were obtained using standardized autobiographical memory questionnaires, such as the Survey of Autobiographical Memory (SAM)^19,20^ or the Questionnaire for the Assessment of Everyday Memory Performance (FEAG)^14,21^. In addition, Dando et al. as well as Li et al. found differences in objective autobiographical memory performance, showing less complete memory reports in aphantasics compared to controls, at least for unfamiliar environments^22,23^. Given his own experiences, Watkins proposes that SDAM may be the most severe form of aphantasia^13^.

Interestingly, this proposal is in line with the episodic memory account by Blomkvist who describes aphantasia as an episodic system condition^24^, building on the constructive episodic simulation hypothesis by Schacter and Addis^25,26^. According to her account, aphantasics are not able to retrieve sensory information (= episodic memory) or (re)combine them to sensory imagery (= mental imagery), either due to a problem with the episodic memory index, the retrieval processes downstream from the memory index or the (re)combination process. This assumption is supported, for example, by findings that future simulations are also impaired in aphantasics, in addition to autobiographical memory^9,10^. Subsequent research found evidence that the memory index situated in the hippocampus^27^ may be impaired in aphantasics, showing aberrant activation and connectivity patterns between the hippocampus and the more downstream visual cortex^11^. It remains unclear, however, why the memory index in aphantasics operates differently from controls.

Importantly, mental imagery has recently been proposed as being a part of a broader inwardly focused cognitive style, reflecting a tendency to prioritize internal experiences and bodily awareness^15,28^. This focus on internal states is closely linked to interoception — the ability to detect and interpret bodily signals — which plays an important role in higher-order cognitive functions related to self-awareness, emotion regulation and the integration of physiological and cognitive processes^29,30^. Theoretical work suggests that interoception serves as the foundation of mental imagery by anchoring it in first-person physiological signals, providing a self-referential perspective and grounding imagery in bodily states. In this view, aphantasia results from a disruption in the integration of interoceptive and sensory information, rather than being merely a deficit in ability to create conscious internal sensory representations^31^.

Moreover, as interoception plays a central role in constructing self-referential experiences, it is also relevant for autobiographical memory, which relies on an embodied perspective to encode and retrieve past events^32^. Consistent with this view, Messina et al. found that individuals with stronger interoceptive awareness recalled more positive memories in a personal rather than public context, reinforcing the idea that interoception contributes to the emotional and self-referential nature of memory^33^. Additionally, interoception shapes the emotional tone of memories over time as individuals with higher interoceptive sensibility exhibit a stronger *fading affect bias*, recalling past events with reduced negative emotional intensity^34^. Individuals with higher interoceptive sensitivity also exhibit better retention by reducing interference during learning^35^, likely by enhancing attention to emotionally salient experiences, particularly positive ones, which are central to self-identity and more resistant to interference^36^. This aligns with the self-memory system hypothesis^37^, which suggests that autobiographical memory promotes self-coherence by prioritizing positive experiences. Neuroimaging evidence further supports the link between interoception and autobiographical memory as they share neural substrates, particularly the insular cortex^29,38^, the major hub for interoceptive processing^30^. For example, emotionally intense memory recall is associated with heightened insular activity^39^, with more vivid positive memories eliciting stronger activation^40^.

In the present study, we examined the relationship between subjective interoception, mental imagery vividness, and self-reported autobiographical memory in a combined sample of individuals with and without aphantasia. To assess subjective interoception, we used complementary measures: the Multidimensional Assessment of Interoceptive Awareness (MAIA) which captures awareness, trust, and regulation of bodily signals in relation to emotional and cognitive processes^41^; the Interoceptive Attention Scale (IATS), which measures attention to internal bodily signals^42^; and the Interoceptive Accuracy Scale (IAS), which measures perceived accuracy of detecting bodily signals^43^. Mental imagery vividness was assessed with the Plymouth Sensory Imagery Questionnaire (PSIQ)^44^, and autobiographical memory was assessed with the Survey of Autobiographical Memory (SAM)^19^, which allowed for a larger sample size than would have been possible using the Autobiographical Interview, which has been used in previous studies^9–11^.

We started by examining whether reduced mental imagery vividness is associated with reduced subjective interoception, which may in turn be the reason for the diminished autobiographical memory in aphantasics, since interoception has been shown to play an important role in autobiographical memory, particularly in shaping its emotional and self-referential aspects. Next, we investigated whether reduced interoceptive awareness was linked to diminished autobiographical memory in our sample and whether this relationship was mediated by imagery vividness, such that imagery functions as a key mechanism linking bodily awareness to memory detail. To address recent concerns that aphantasia (= complete absence of mental imagery) and hypophantasia (= reduced mental imagery)^45^ may represent qualitatively distinct subgroups^46^, we differentiated core aphantasia from hypophantasia by comparing them on interoceptive and memory measures, before following up with dimensional analyses.

## Methods

### Participants

**Fig. 1:**
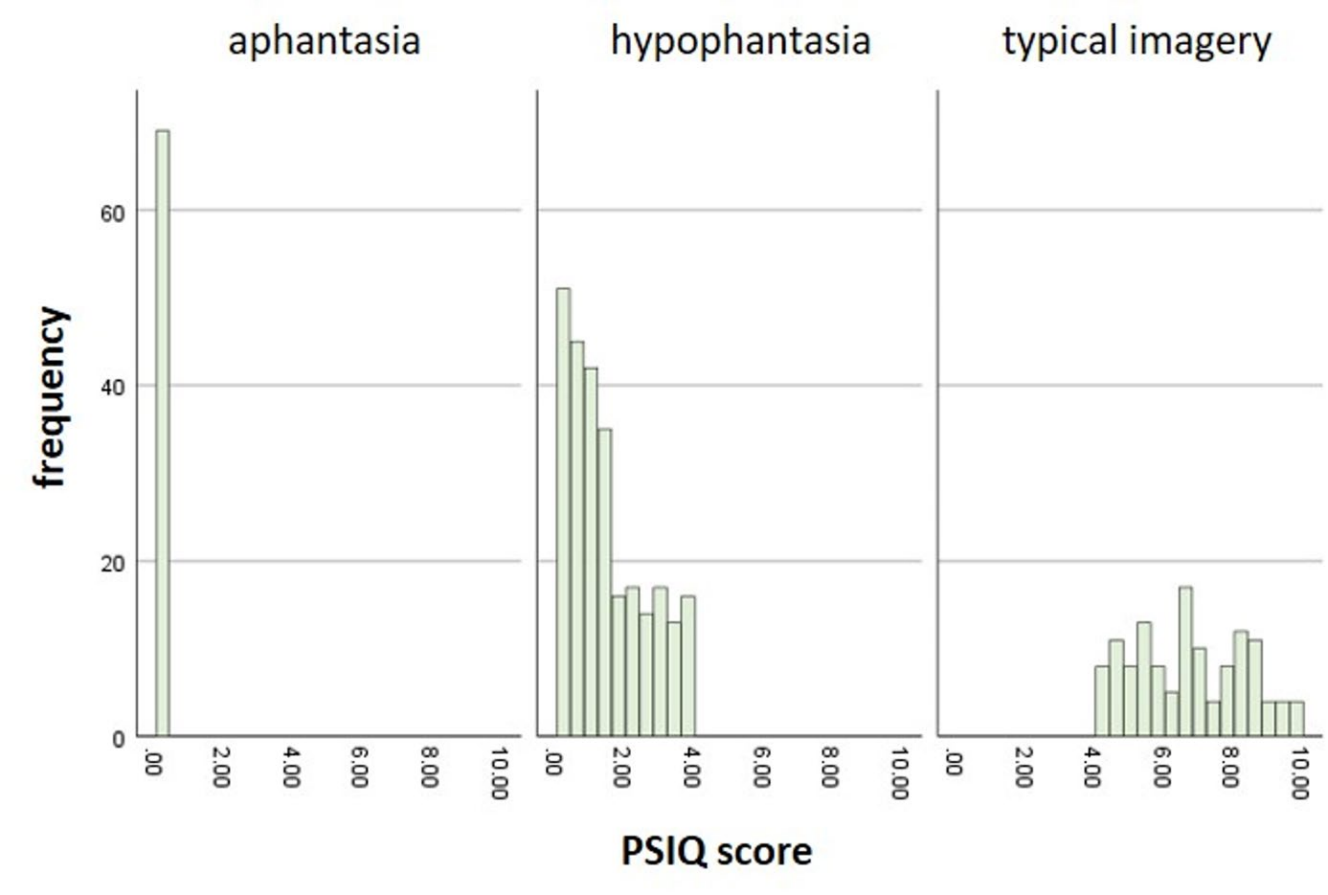
Distribution of PSIQ scores separated by group.

Participants were recruited by advertising the study on the University of Surrey campus, as well as via the *Aphantasia Research Database Bonn*^8,14,47^ in order to also reach individuals with aphantasia. Ethical approval was obtained from the Ethics Committee of the University of Surrey. The final sample consisted of 468 participants, from which 69 were core aphantasics (PSIQ = 0), 266 were hypophantasics (PSIQ > 0 and PSIQ ≤ 4) and 133 were imagers (PSIQ > 4). A distribution of PSIQ scores separated by group is depicted in Fig. 1. Since some participants cancelled the questionnaires early, there are deviations from these numbers in individual analyses. For the SAM, 15 participants were missing, for the IAS, 22 participants were missing, for the IATS, 30 participants were missing, and for the MAIA subscales, 53 to 55 participants were missing. Thus, each analysis was performed with a minimum of 413 participants. There were no systematic group differences in the time of discontinuation, *F*(2, 465) = 0.48, *p* = .618. On average, participants were *M* = 40.34 (*SD* = 14.55) years old. 36.1% participants were male, 59.6% were female and 4.3% reported another gender. On average, hypophantasics were 5.61 years younger than aphantasics (*p* = .013), but there were no significant differences between hypophantasics and typical imagers (*p* = .178) as well as between aphantasics and typical imagers (*p* = .929). Moreover, in the hypophantasic group, there were significantly fewer male participants in favor of female participants (*p* < .05).

### Materials and procedure

After the participants had given their informed consent and answered some basic demographic questions (i.e., age and gender), they were asked to complete several questionnaires, either in English or in German, according to their choice. First, they completed the Plymouth Sensory Imagery Questionnaire (PSIQ), which is a 35 item questionnaire measuring the vividness of mental imagery in the following modalities on a 5-point Likert scale from ‘no image at all’ to ‘image as clear and vivid as real life’: vision, sound, smell, taste, touch, bodily sensation, and emotional feeling. Each modality comprises 5 items, each describing a certain sensory experience which participants are asked to imagine (e.g., ‘Imagine the appearance of a sunset.’)^44^. Next, participants answered the episodic memory subscale of the Survey of Autobiographical Memory (SAM), which comprises 8 items on a 5-point Likert scale from ‘strongly disagree’ to ‘agree strongly’. Sample items are ‘I am highly confident in my ability to remember past events’ and ‘When I remember events, I remember a lot of details’^19^. Afterwards, participants answered the Interoceptive Accuracy Scale (IAS)^43^ as well as the Interoceptive Attention Scale (IATS)^42^, each comprising 21 items on a 5-point Likert scale from ‘disagree strongly’ to ‘strongly agree’. Sample items are ‘I can always accurately perceive when I am hungry’ and ‘Most of the time my attention is focused on whether I am hungry’, respectively. Last, participants answered the Multidimensional Assessment of Interoceptive Awareness 2 (MAIA), which comprises 37 items on a 5-point Likert scale from ‘never’ to ‘always’^41^. The MAIA items are aggregated into the subscales noticing, not-distracting, not-worrying, attention regulation, emotional awareness, self-regulation, body listening and trusting. The SAM was aggregated to an average score according to Setton et al.^48^.

### Statistical analyses

First, we calculated a MANOVA with group allocation (core aphantasia vs. hypophantasia vs. imagers) as independent variable and scores of the IAS, IATS, MAIA and SAM subscales as dependent variables to assess differences in interoceptive abilities as well as episodic memory between the groups. Next, we examined correlations between the total PSIQ score and the IAS, IATS, MAIA and SAM subscales to identify the most important associations with the vividness of mental imagery. This was followed up by a hierarchical regression to examine the incremental validity of the IAS, IATS and MAIA subscales in predicting self-reported episodic memory. Last, we included the significant variables from the hierarchical regression analysis in a structural model predicting episodic memory, using mental imagery vividness as a mediator, to examine whether the association between a lack of interoceptive abilities and impairments in episodic memory is mediated by mental imagery. The mediation analyses were conducted with Jamovi (Version 2.6), using a series of ordinary least squares (OLS) regressions to estimate direct, indirect, and total effects. We report standardized beta coefficients as well as p-values. The rest of the analyses were performed using SPSS (Version 29.0).

## Results

### Differences in interoceptive abilities

The MANOVA was significant, with the most conservative result being obtained using Pillai’s trace, *V* = 0.42, *F*(22, 802) = 9.63, *p* < .001, η_p_^2^ = 0.21. Follow-up ANOVAs are depicted in Table 1, revealing significant differences for all dependent variables except the MAIA subscales not-distracting and not-worrying.^1^ Biggest differences were obtained for episodic memory (η_p_^2^ = 0.25), followed by emotional awareness (η_p_^2^ = 0.12) and body listening (η_p_^2^ = 0.11). Tukey-HSD post-hoc tests revealed that most of the effects were based on significant differences between core aphantasia/hypophantasia and imagers. Differences between core aphantasia and hypophantasia were only found for IATS and emotional awareness.

**Table 1:** Results of ANOVAs comparing core aphantasics, hypophantasics and imagers (*N* = 413).

	core aphantasia (*n* = 60)		hypophantasia (*n* = 233)		imagers (*n* = 120)				
Dependent variable	*M*	*SD*	*M*	*SD*	*M*	*SD*	*F*(2, 410)	*p*	η_p_^2^
*SAM*									
episodic memory	1.85^a^	0.64	1.97^a^	0.68	2.92^b^	0.96	40.72	< 0.001	0.25
IAS	4.03	0.56	3.87	0.61	3.99	0.59	0.94	0.073	0.01
IATS	1.71^a^	0.57	1.97^b^	0.65	2.26^c^	0.82	6.77	< 0.001	0.07
*MAIA*									
noticing	3.44^a^	1.29	3.54^a^	1.01	4.13^b^	1.00	15.96	< 0.001	0.07
not-distracting	3.14	0.90	3.00	0.88	2.97	0.96	0.62	0.471	0.00
not-worrying	3.82	1.17	3.50	0.99	3.45	0.91	2.93	0.054	0.01
attention regulation	3.08^a^	1.15	3.16^a^	1.06	3.84^b^	1.12	20.65	< 0.001	0.08
emotional awareness	2.90^a^	1.30	3.59^b^	1.21	4.26^c^	1.01	39.30	< 0.001	0.12
self-regulation	3.03^a^	1.30	2.97^a^	1.23	3.70^b^	1.22	22.35	< 0.001	0.07
body listening	2.49^a^	1.19	2.82^a^	1.10	3.58^b^	1.21	31.49	< 0.001	0.11
trusting	3.80^a^	1.31	3.86^a^	1.27	4.33^b^	1.22	9.92	0.002	0.03

Note. Different superscripts indicate significant results in Tukey-HSD post-hoc tests (*p* < .05). SAM = Survey of Autobiographical Memory, IAS = Interoceptive Accuracy Scale, IATS = Interoceptive Attention Scale, MAIA = Multivariate Assessment of Interoceptive Abilities.

### Correlations with mental imagery vividness

The correlations between mental imagery vividness and interoceptive abilities as well as episodic autobiographical memory are depicted in Table 2. As expected from the previous analyses, correlations with mental imagery vividness were highest for episodic autobiographical memory (*r* = .55), emotional awareness (*r* = .36) and body listening (*r* = .39).

**Table 2:** Descriptive statistics and correlations for study variables (*N* = 468).

Variable	*n*	*M*	*SD*	α	1	2	3	4	5	6	7	8	9	10	11
1. PSIQ	468	2.77	2.90	0.99	—										
2. SAM	453	2.23	0.88	0.85	0.55***	—									
3. IAS	446	3.93	0.59	0.90	**0.08**	0.13**	—								
4. IATS	438	2.02	0.71	0.93	0.24***	0.15**	-0.03	—							
5. MAIA noticing	414	3.70	1.09	0.73	0.30***	0.29***	0.41***	0.24***	—						
6. MAIA not-distracting	415	3.01	0.90	0.77	** − 0.03**	0.02	0.19***	** − **0.15**	-0.03	—					
7. MAIA not-worrying	414	3.53	1.00	0.73	** − 0.05**	** − **0.05	0.07	** − **0.23***	-0.08	-0.02	—				
8. MAIA attention regulation	415	3.35	1.13	0.88	0.32***	0.24***	0.39***	0.10	0.68***	0.02	0.24***	—			
9. MAIA emotional awareness	414	3.69	1.25	0.85	0.36***	0.29***	0.33***	0.24***	0.72***	** − **0.04	** − **0.11*	0.56***	—		
10. MAIA self-regulation	415	3.19	1.28	0.85	0.27***	0.18***	0.29***	0.08	0.57***	0.03	0.22***	0.75***	0.55***	—	
11. MAIA body listening	413	3.00	1.21	0.82	0.39***	0.27***	0.31***	0.25***	0.67***	0.05	0.03	0.66***	0.68***	0.65***	—
12. MAIA trusting	414	3.99	1.28	0.82	0.20***	0.16***	0.37***	0.02	0.46***	0.14**	0.33***	0.61***	0.38***	0.57***	0.52***

Note. PSIQ = Plymouth Sensory Imagery Questionnaire (range 0–10), SAM = Survey of Autobiographical Memory (range 0–5), IAS = Interoceptive Accuracy Scale (range 0–5), IATS = Interoceptive Attention Scale (range 0–5), MAIA = Multivariate Assessment of Interoceptive Abilities (range 0–5). Differences in n are due to missing scale values. Correlations with mental imagery are depicted in bold. *p < .05. **p < .01. ***p < .001.

### Predicting autobiographical memory impairments

**Fig. 2:**
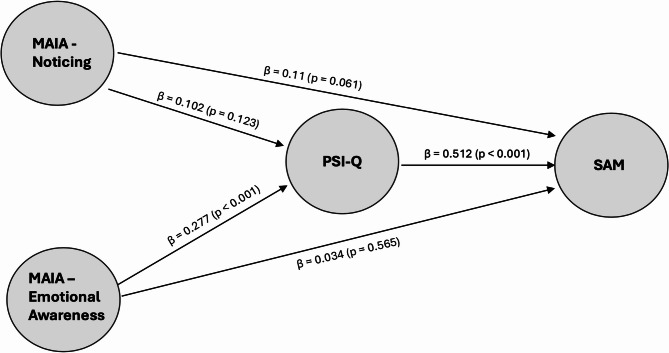
Standardized direct path coefficients from the GLM-based mediation model (*N* = 414). The model included two interoceptive predictors from the Multidimensional Assessment of Interoceptive Awareness (MAIA): Emotional Awareness and Noticing. PSI-Q = Plymouth Sensory Imagery Questionnaire, SAM = Survey of Autobiographical Memory.

Next, we performed a hierarchical regression analysis, including all interoceptive ability measures (i.e., all MAIA subscales as well as IAS and IATS). The analysis revealed that the MAIA subscales emotional awareness and noticing were the only subscales adding unique variance in predicting self-reported autobiographical memory (see Table 3). Thus, we decided to include these variables in our structural model to investigate whether mental imagery vividness mediates the association between a lack of interoceptive abilities and impairments in episodic memory (see Fig. 2).

**Table 3:** Hierarchical regression analysis of interoceptive abilities on self-reported autobiographical memory (*N* = 413).

Variable	β	*t*	*p*	*ΔF*	*R*_*corr*_^*2*^	*ΔR*_*corr*_^*2*^
*Step 1*						
MAIA emotional awareness	0.29	6.24	< 0.001	38.92	0.08	0.08
*Step 2*						
MAIA emotional awareness	0.18	2.61	0.009	5.72	0.10	0.02
MAIA noticing	0.16	2.39	0.017			

Note. MAIA = Multidimensional Assessment of Interoceptive Abilities.

Both variables showed significant total effects on autobiographical memory (emotional awareness: β = 0.18, 95% CI [0.03, 0.22], *p* = .009; noticing: β = 0.16, 95% CI [0.02, 0.24], *p* = .02). The indirect effect of emotional awareness via imagery was significant (β = 0.14, 95% CI [0.05, 0.15], *p* < .001), while the direct effect was nonsignificant (β = 0.03, 95% CI [− 0.06, 0.11], *p* = .565), consistent with full mediation. In contrast, the indirect effect of noticing through imagery was not statistically significant (β = 0.05, 95% CI [− 0.01, 0.10], *p* = .123), and the direct effect was marginal (β = 0.11, 95% CI [− 0.01, 0.18], *p* = .06). These results suggest that the MAIA emotional awareness subscale influences autobiographical memory primarily through imagery, whereas the relationship between the noticing subscale and SAM is less clearly mediated and may involve additional pathways.

## Discussion

Our findings demonstrate that subjectively reported interoceptive abilities are closely linked to mental imagery vividness and self-reported autobiographical memory. Core aphantasics and hypophantasics exhibited significantly lower scores in episodic memory, interoceptive attention and on various subscales of the MAIA questionnaire, including emotional awareness and noticing, indicating that reduced imagery ability is associated with weaker interoceptive processing. Univariate correlations confirmed that episodic memory, interoceptive abilities and mental imagery were associated, suggesting that interoceptive processes contribute to mental imagery ability and autobiographical memory. To check for unique variance predicting autobiographical memory, we performed a multivariate hierarchical regression analysis, showing that the MAIA subscales emotional awareness and noticing were the only unique significant predictors of autobiographical memory. Mediation analyses revealed that mental imagery ability plays a key role in linking emotional awareness to autobiographical memory, but not noticing. These results suggest that emotional awareness may influence memory through imagery-based mechanisms, whereas noticing may contribute more directly, possibly by increasing attentional access to bodily signals during recall. This highlights that different interoceptive dimensions may relate to memory via distinct cognitive pathways — some involving imagery, others potentially involving more immediate awareness of bodily states. This interpretation aligns with evidenced discussed in the introduction that interoception contributes to autobiographical memory through multiple mechanism (including emotional salience, attentional focus, and embodied self-relevance), of which not all include mental imagery.

### Subjective interoception and aphantasia: MAIA, IATS and IAS

MAIA, IAS and IATS all assess aspects of interoceptive processing, though they focus on distinct dimensions. MAIA is focused on capturing how individuals subjectively perceive and interpret their bodily sensations across various domains, such as emotional awareness, attention regulation, and self-trust. It emphasizes cognitive and emotional engagement with bodily signals, assessing how individuals integrate interoception into their sense of self and well-being. In contrast, the IAS and IATS assess how confident individuals feel in their ability to detect (and how much attention they allocate to) more basic bodily sensations such as hunger, heartbeat, and pain without external cues. IATS and the MAIA subscales noticing, attention regulation, emotional awareness, self-regulation, body listening and trusting were moderately correlated with mental imagery. However, we found no evidence for a significant relationship between mental imagery and the IAS or between mental imagery and the MAIA subcales non-distracting and not-worrying.

The distinction between MAIA and IATS on the one hand and IAS on the other hand may arise because MAIA and IATS measure active engagement with bodily signals, meaning they assess how much individuals attend to, interpret, and incorporate bodily sensations into their cognitive and emotional processes. Unlike IAS, which focuses on whether individuals believe they can detect bodily signals, MAIA captures the extent to which individuals consciously notice and respond to internal sensations, such as recognizing changes in breathing, bodily tension, or emotional states. Similarly, IATS reflects the degree of attentional focus allocated to bodily signals, indicating how much individuals habitually monitor and tune into their interoceptive experiences.

This active engagement is particularly evident in the MAIA subscales emotional awareness and body listening, which were most strongly associated with mental imagery and assess how interoceptive signals are used in self-regulation, decision-making, and emotional processing. Emotional awareness measures the ability to link bodily sensations with emotional experiences, such as recognizing physical tension during stress or feeling bodily relaxation during positive emotions. Body listening reflects a deeper introspective connection with the body, assessing whether individuals use bodily sensations as cues to guide their emotions and behavior. By measuring how interoceptive signals are actively attended to and utilized, MAIA and IATS are more directly relevant to mental imagery, which similarly requires individuals to internally focus on and manipulate sensory and emotional information. Our results also indicate that core aphantasics and hypophantasics do not seem to be qualitatively different with regard to interoceptive abilities^46^. This may indicate that a certain level of mental imagery is necessary for successful interoception, which is not reached even by hypophantasics.

### The role of subjective interoception in autobiographical deficits in aphantasia

Our results are consistent with the view that subjective interoceptive awareness plays an important role in autobiographical memory and that deficits observed in aphantasia reflect this relationship. The ability to reconstruct past experiences vividly depends not only on sensory recall but also on the integration of interoceptive and emotional signals, which help anchor memories to the self^33,35,36,49^. Our findings support this idea, as individuals with weaker self-reported interoceptive awareness — particularly in emotional awareness — also showed reduced mental imagery vividness and autobiographical memory recall. This aligns with theoretical models suggesting that interoception provides the physiological foundation for self-referential cognition, allowing memories to be experienced in a first-person, embodied perspective^31^. Noticing, on the other hand, seems to contribute to autobiographical memory independent from mental imagery, possibly by directly increasing attentional access to bodily signals during recall.

Importantly, the SAM is a self-report measure assessing perceived recollective capacity that is subject to introspection and memory confidence. We used this scale to achieve a higher sample size than would have been possible with more extensive methods like the Autobiographical Interview. While the SAM is highly reliable, there are only weak correlations between its episodic subscale and reported internal details in the Autobiographical Interview and high correlations with memory vividness and self-efficacy^48^, indicating that the SAM rather measures confidence in episodic memory rather than episodic memory itself. Nevertheless, an interesting pattern emerged, showing that only emotional awareness and noticing were predictive for self-perceived recollective capacities and that only the relationship between emotional awareness and SAM was mediated by mental imagery. This could mean that both noticing and the emotional interpretation of bodily signals is necessary for memory confidence, but that the interpretation additionally requires a good sensory representation. Future research should investigate whether other aspect of autobiographical memory are also dependent on interoceptive processes, such as recollection of memory details^9–11^ and the completeness and accuracy of autobiographical memory^22,23^.

Overall, the link between subjective interoception and confidence in autobiographical memory may explain why aphantasics report difficulties recalling personal events with the same richness and sensory detail as typical imagers^9–11^. Interoceptive signals contribute to emotional tagging during encoding, shaping how events are later retrieved and integrated into the self^33^. Deficits in interoceptive awareness, as seen in aphantasia, may therefore impair the reinstatement of internal bodily states associated with past experiences, leading to less vivid and emotionally immersive memory recall.

## Data Availability

The data that support the findings of this study are openly available at (https://osf.io/gaj8k/?view_only=81cb53ea255b4ae597801c36a8fd78ae).

## Footnotes

1 A reanalysis with demographically matched groups led to similar results, *V* = 0.50, *F*(22, 462) = 7.03, *p* < .001, η_p_^2^ = 0.25, with the exception of the MAIA subscale “not worrying” also reaching significance, *F*(2, 240) = 4.38, *p* = .013, η_p_^2^ = 0.04.
